# Subconjunctival aflibercept inhibits corneal angiogenesis and VEGFR-3^+^CD11b^+^ cells

**DOI:** 10.1007/s00417-024-06560-4

**Published:** 2024-07-09

**Authors:** Chang Ho Yoon, Jung Hwa Ko, Hyun Ju Lee, Hyun Beom Song, Joo Youn Oh

**Affiliations:** 1 Laboratory of Ocular Regenerative Medicine and Immunology, Biomedical Research Institute, Seoul National University Hospital, 101 Daehak-ro, Jongno-gu, Seoul, 03080 Korea; 2 Department of Ophthalmology, Seoul National University College of Medicine, 103 Daehak-ro, Jongno- gu, Seoul, 03080 Korea; 3 Department of Tropical Medicine and Parasitology, Institute of Endemic Diseases, Seoul National University College of Medicine, 103 Daehak-ro, Jongno-gu, Seoul, 03080 Korea; 4 Department of Biomedical Sciences, Seoul National University College of Medicine, 103 Daehak-ro, Jongno-gu, Seoul, 03080 Korea

**Keywords:** Aflibercept, Angiogenesis, Cornea, Macrophage, Monocyte, Vascular endothelial growth factor receptor

## Abstract

**Purpose:**

This study aimed to investigate the effects of subconjunctival injection of aflibercept, a soluble protein decoy for VEGFR-1 and VEGFR-2, on corneal angiogenesis and VEGFR-expressing CD11b^+^ cells in a mouse model of suture-induced corneal neovascularization.

**Methods:**

Corneal neovascularization was induced in BALB/c mice by placing three sutures on the cornea. Immediately after surgery, either 200 µg aflibercept (5 µL) or an equal volume of phosphate-buffered saline (PBS) was administered into the subconjunctival space. Seven days after later, corneal new vessels were quantified through clinical examination and measurement of the CD31-stained area in corneal flat mounts. The levels of pro-angiogenic and inflammatory markers in the cornea were evaluated using RT-qPCR. The percentages of VEGFR-2^+^CD11b^+^ cells and VEGFR-3^+^CD11b^+^ cells were analyzed in the cornea, blood, and draining cervical lymph nodes (DLNs) using flow cytometry.

**Results:**

Subconjunctival injection of aflibercept significantly reduced the growth of corneal new vessels compared to subconjunctival PBS injection. The mRNA levels of *Cd31*, vascular growth factors (*Vegfc* and *Angpt1*), and pro-angiogenic/inflammatory markers (*Tek/Tie2*, *Mrc1*, *Mrc2*, and *Il6*) in the cornea were downregulated by subconjunctival aflibercept. Also, the percentage of VEGFR-3^+^CD11b^+^ cells in the cornea, blood, and DLNs was decreased by aflibercept, whereas that of VEGFR-2^+^CD11b^+^ cells was unaffected.

**Conclusion:**

Subconjunctival aflibercept administration inhibits inflammatory angiogenesis in the cornea and reduces the numbers of cornea-infiltrating and circulating VEGFR-3^+^CD11b^+^ cells.

**Supplementary Information:**

The online version contains supplementary material available at 10.1007/s00417-024-06560-4.

## Key messages

***What is known:***

• Aflibercept inhibits hemangiogenesis in the eye by interfering with the VEGF-A/VEGFR-2 signaling.

***What is new:***

• Subconjunctival aflibercept downregulates the VEGF-C and angiopoietin signaling in the cornea.

• Subconjunctival aflibercept reduces both circulating and cornea-infiltrating VEGFR-3^+^CD11b^+^ myeloid cells.

**Supplementary Information:**

The online version contains supplementary material available at 10.1007/s00417-024-06560-4.

## Introduction

Aflibercept, also termed a vascular endothelial growth factor (VEGF) trap, is a recombinant fusion protein that acts as a soluble protein decoy for VEGF receptors, VEGFR-1 and VEGFR-2. Since it binds to VEGF-A with a higher affinity than its natural competitors (VEGFR-1 and VEGFR-2), aflibercept inhibits VEGF signaling and thus prevents pathologic angiogenesis [1, 2]. Based on its anti-angiogenic activity, intravitreal injection of aflibercept (Eylea®, Regeneron Pharmaceuticals, Inc., Tarrytown, NY) has been approved by FDA for treatment of retinal and choroidal neovascularization such as neovascular age-related macular degeneration, myopic choroidal neovascularization, macular edema associated with retinal vein occlusion, diabetic macular edema, and diabetic retinopathy. Especially, aflibercept has been shown to have markedly higher affinity for VEGF compared to bevacizumab and ranibizumab, widely-used monoclonal antibodies against VEGF-A [3, 4].

The cornea is devoid of both blood and lymphatic vessels and maintains its avascularity through immunologic and angiogenic privilege. An injury to the cornea can cause inflammation and disrupt its immune and angiogenic privilege, thereby leading to the growth of corneal new vessels and loss of corneal transparency [5, 6]. The mechanisms of corneal neovascularization involve both hemangiogenesis (driven by VEGF-A binding to VEGFR-1 and VEGFR-2) and lymphangiogenesis (driven by VEGF-C and VDGF-D binding to VEGFR-3) [5–7], Therefore, it is possible that therapies targeting VEGFs and their receptors are effective in attenuating corneal neovascularization as well as choroidal and retinal neovascular diseases. Indeed, previous studies by our group and others reported the inhibitory effects of topical, subconjunctival, or intracorneal administration of bevacizumab and ranibizumab on corneal neovascularization in animal models and human clinical studies [8–15]. In recent years, several studies have demonstrated superior or similar efficacies of aflibercept, a more potent VEGF-A inhibitor, in reducing corneal neovascularization compared to bevacizumab [16–18].

In this study, we investigated the effects of subconjunctival aflibercept injection on corneal angiogenesis in a model of suture-induced inflammatory corneal neovascularization. Specifically, we analyzed the effects of aflibercept on corneal new vessel growth, the levels of pro-angiogenic and inflammatory monocyte/macrophage markers in the cornea, and the numbers of circulating and cornea-infiltrating VEGFR-expressing myeloid cells.

## Methods

### Animal model and treatment

The experimental protocol was approved from the Institutional Animal Care and Use Committee of Seoul National University Hospital Biomedical Research Institute (Seoul, Korea) and followed the guidelines stated by ARVO for ophthalmic and vision research.

Eight-week-old male BALB/c mice (KOATECH, Pyeongtaek, Korea) were used for the study. Under anesthesia with intraperitoneal injection of zolazepam-tiletamine (Zoletil®, Virbac, Carros, France) and topical administration of 0.5% proparacaine hydrochloride ophthalmic solution (Hanmi Pharm Co., Ltd., Seoul, Korea), the cornea was marked using a 2-mm-diameter trephine, and three 10 − 0 nylon sutures were evenly placed 120° apart from each other, through the epithelial and stromal layers, and with both points of each stitch going in and out of the cornea along the mark. The knots were left unburied.

Immediately after the placement of corneal sutures, either 200 µg aflibercept (5 µL) (Eylea®, Regeneron Pharmaceuticals, Inc.) or the same volume of phosphate-buffered saline (PBS) was injected subconjunctivally using a 33-gauge needle (Hamilton, Reno, NV). Seven days later, the mice were subjected to assays.

### Clinical examination

Corneal new vessels were examined under slit-lamp biomicroscopy and photographed with a camera mounted on a microscope. The extent of corneal neovascularization was graded independently by two individuals (C.H.Y. and J.Y.O.) in a blinded manner using the standardized scale system: the growth of corneal new vessels was graded from 0 to 3 in each quadrant of the cornea, and scores for each quadrant were summed to obtain the clinical score (range 0 to 12) for each eye [19–21].

### Histopathology

The corneas were extracted and fixed in 4% paraformaldehyde overnight at 4˚C. After washing with PBS, the tissues were treated with proteinase K for 5 min at room temperature, followed by treatment with methanol for 30 min. After PBS washing, the tissues were blocked with 2% BSA overnight at 4˚C, and then were incubated with primary antibodies against CD31 (BD Pharmingen, San Diego, CA), CD11b (BD Pharmingen), VEGFR-2 (R&D Systems, Minneapolis, MN), or VEGFR-3 (R&D Systems) for 16 h at 4˚C. The next day, the corneas were incubated with secondary antibodies overnight at 4˚C in the dark. The stained corneas were flat-mounted onto slides and examined under a fluorescence microscope (Nikon, Tokyo, Japan).

For quantification of the CD31-stained area, the digital fluorescence images of corneal flat mounts were analyzed using ImageJ software (National Institutes of Health, Bethesda, MD) [20, 22]. The total area of the cornea was manually delineated by outlining the innermost vessels of the limbal arcade and removing areas outside the delineated margin. After background subtraction and thresholding, the percentage of CD31^+^ area out of the total corneal area was calculated.

For quantification of VEGFR-2^+^CD11b^+^ or VEGFR-3^+^CD11b^+^ myeloid cells in the cornea, the numbers of VEGFR-2^+^CD11b^+^ and VEGFR-3^+^CD11b^+^ cells were counted in the regions adjacent to the limbus along the sutures of the cornea under a fluorescence microscope, at a magnification of X400. For each quantification, the corneal stroma with approximate size of 280 μm X 450 μm was covered.

### Real-time reverse transcription quantitative polymerase chain reaction (RT-qPCR)

The corneal tissue was dissected into small pieces using microscissors, lysed in RNA isolation reagent (RNA Bee, Tel-Test, Friendswood, TX), and homogenized using an ultrasound sonicator (Ultrasonic Processor, Cole Parmer Instruments, Vernon Hills, IL). Total RNA was extracted from the lysates using the RNeasy Mini kit (Qiagen, Valencia, CA) and converted to first-strand cDNA by reverse transcription using the High Capacity RNA-to-cDNA™ Kit (Applied Biosystems, Carlsbad, CA). RT-qPCR amplification was performed using TaqMan® Universal PCR Master Mix (Applied Biosystems) and specific TaqMan® probe sets for *Cd31*, *Vegfc*, *Angpt1* (angiopoietin-1), *Vegfa*, *Tek/Tie2*, *Mrc1* (mannose receptor C-type 1), *Mrc2*, and *Il6* (all from Applied Biosystems) in an automated instrument (ABI 7500 Real Time PCR System, Applied Biosystems). Assay ID for each TaqMan®probe was as follows: Mm01242576_m1 for *Cd31*; Mm00456503_m1 for *Angpt1*; Mm00437306_m1 for *Vegfa;* Mm00437310_m1 for *Vegfc*; Mm00443243_m1 for *Tek/Tie2*; Mm00485148_m1 for *Mrc1*; Mm00485184_m1 for *Mrc2*; Mm00446190_m1 for *Il6.* The data obtained were normalized to *Gapdh* (Mm99999915_g1) and expressed as fold changes relative to the controls.

### Flow cytometry

Whole blood was collected using a 26-gauge needle with a heparin-precoated syringe via cardiac puncture and preserved in EDTA tube. The collected blood was treated with red blood cell lysis buffer (BD Biosciences, San Diego, CA) for 5 min and centrifuged at 2,000 rpm for 10 min. Ocular draining cervical lymph nodes (DLNs) were minced into small pieces between the frosted ends of two glass slides in RPMI 1640 medium (WelGENE, Daegu, Korea) containing 10% fetal bovine serum (Gibco, Carlsbad, CA) and 1% penicillin-streptomycin (Gibco) on ice. The resultant single-cell suspension from the blood and DLNs were stained with fluorescence-conjugated antibodies against CD11b-FITC, VEGFR-2-APC, and VEGFR-3-PE (all from eBioscience, San Diego, CA) for 30 min at 4˚C. The stained cells were analyzed using the S1000EXi Flow Cytometer (Stratedigm, San Jose, CA), and the obtained data were analyzed using the FlowJo program (Tree Star, Ashland, OR).

### Statistical analysis

Prism software (GraphPad, San Diego, CA) was used for statistical tests and generation of graphs. The Shapiro-Wilk test or Kolmogorov-Smirnov test was used to determine a normal distribution of data in each group. The unpaired *t*-test or Mann-Whitney test was used for comparisons of mean values between two groups. One-way ANOVA followed by Tukey’s Honestly Significant Difference test or Kruskal–Wallis test followed by Dunn’s multiple-comparisons test was employed for comparisons of the means between more than two groups. The data were presented as mean ± SD. Differences were considered significant at *p* < 0.05.

## Results

### Subconjunctival aflibercept inhibits corneal new vessel growth

**Fig. 1:**
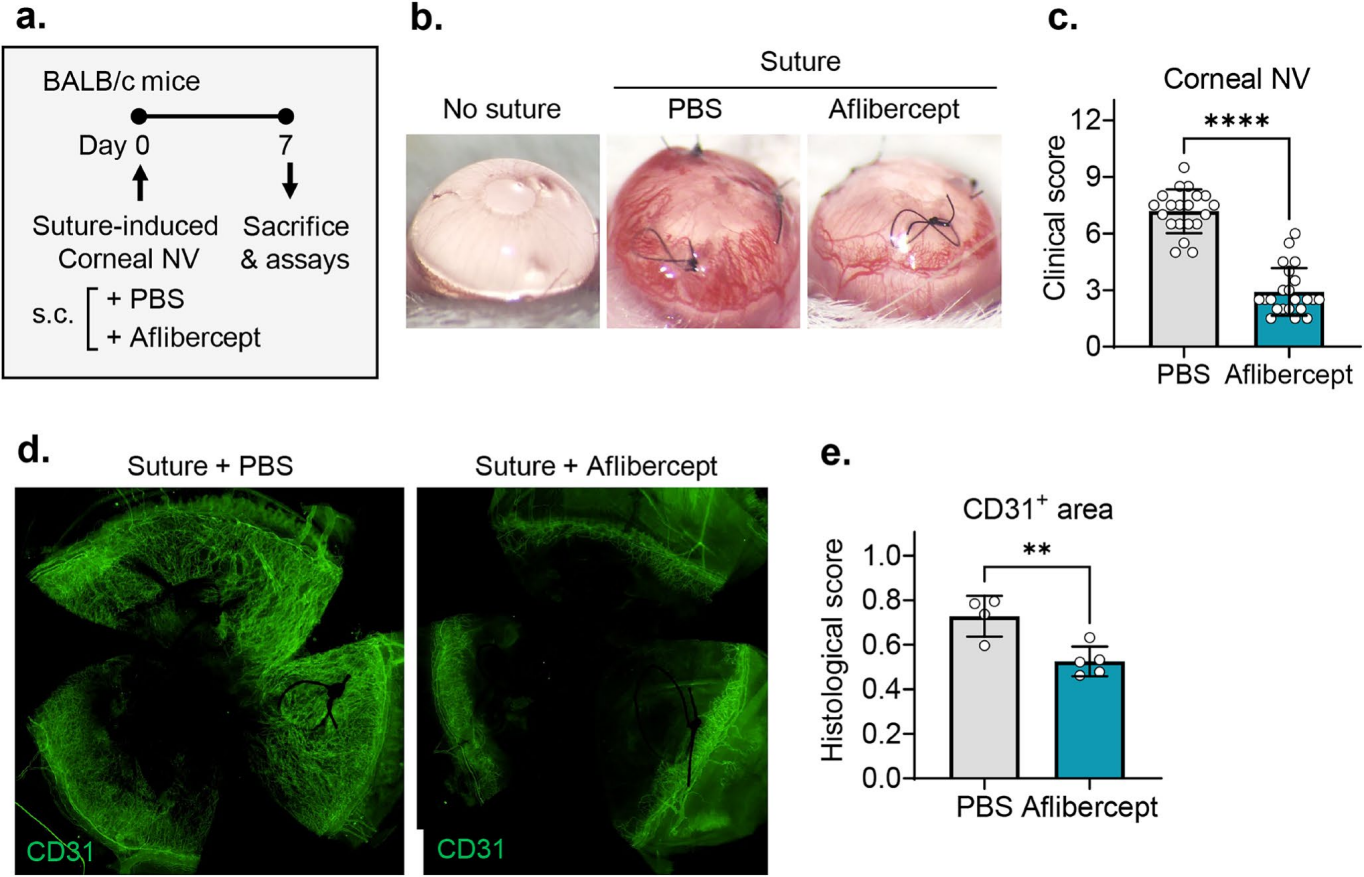
Subconjunctival aflibercept attenuates corneal neovascularization. **a** Experimental scheme. Corneal sutures were applied in BALB/c mice for induction of corneal neovascularization, and aflibercept (200 µg in 5 µL) (Eylea®, Regeneron Pharmaceuticals, Inc., Tarrytown, NY) or PBS (5 µL) was subconjunctivally injected. Seven days later, the corneas were clinically observed, and the corneas, blood and DLNs were collected for assays. **b, c** Representative corneal photographs (**b**) and quantification of corneal new vessels as graded by the standardized scoring system (**c**). **d, e** Representative microphotographs of whole-corneal flat mounts with CD31 immunostaining (**d**) and quantification of CD31-stained area (**e**). Mean values ± SD are shown from three independent experiments. Each circle depicts the data from an individual eye. **p < 0.01, ****p < 0.0001, as analyzed by Mann-Whitney test (**c**) or by unpaired *t*-test (**e**)

To induce inflammatory corneal neovascularization, we applied three 10 − 0 nylon sutures intrastromally onto the cornea of BALB/c mice. For treatment, we injected either 200 µg aflibercept (5 µL) or an equal volume of PBS (5 µL) into the subconjunctival space. Seven days later, the cornea was examined and extracted for histological and molecular assays, and the blood and DLNs were collected for flow cytometric analysis (Fig. 1a).

Consistent with previous observations [20], corneal sutures induced the growth of new vessels from the limbal arcade toward the corneal center, as examined by slit-lamp biomicroscopy (Fig. 1b). Subconjunctival aflibercept injection markedly reduced the extent of corneal neovascularization (Fig. 1b). Clinical scores of corneal neovascularization were significantly lower in aflibercept-treated eyes compared to those treated with PBS (Fig. 1c). These clinical findings were confirmed by CD31 staining of corneal whole mounts. The CD31-stained area was significantly smaller in the aflibercept-treated corneas compared to the PBS-treated controls (Fig. 1d, e).

### Subconjunctival aflibercept downregulates pro-angiogenic and inflammatory molecules in the cornea

**Fig. 2:**
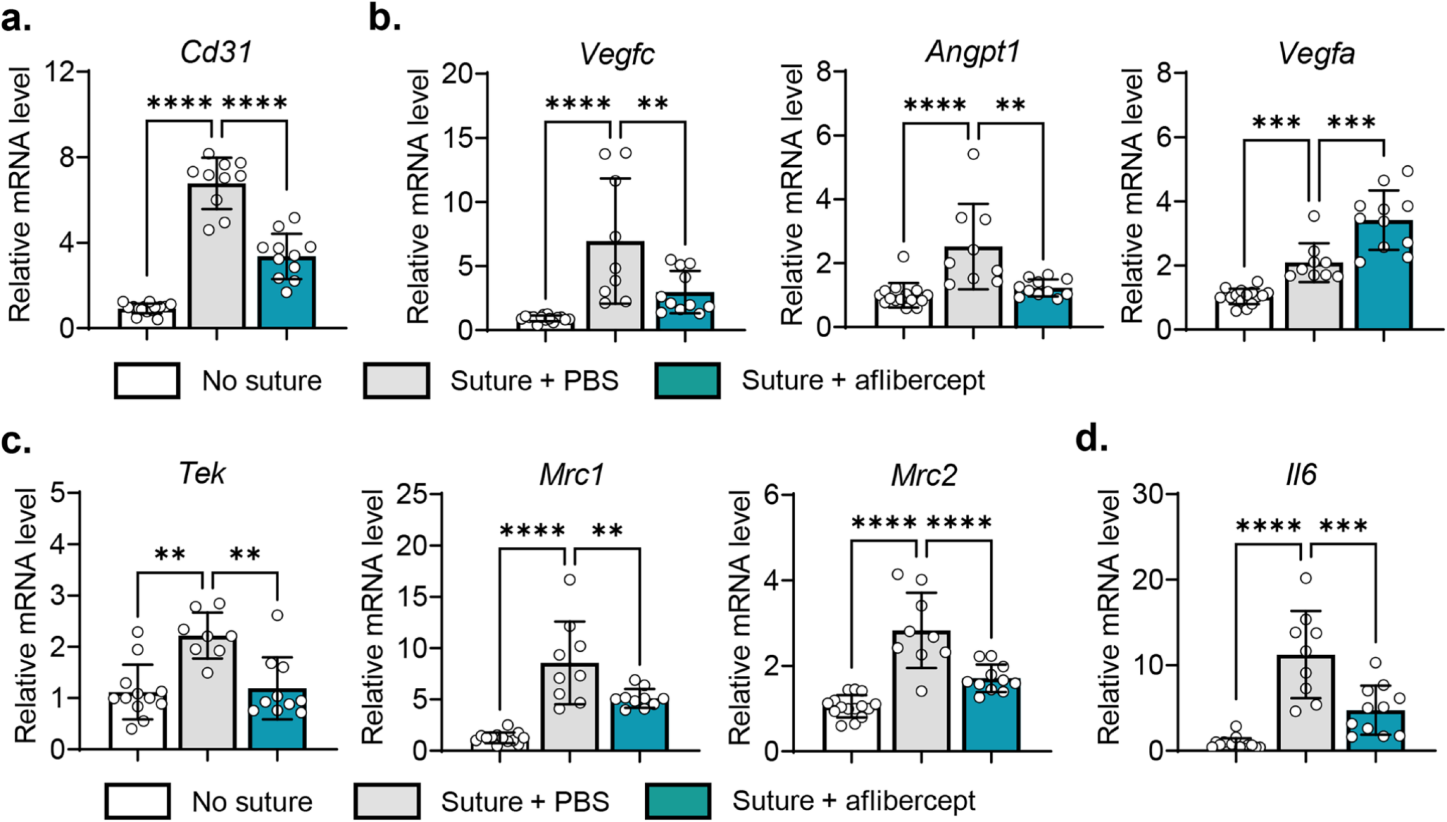
Subconjunctival aflibercept suppresses the expression of pro-angiogenic and inflammatory molecules in the cornea. **a, b, c, d** RT-qPCR for *Cd31* (**a**), vascular growth factors (*Vegfc* and *Angpt1*) (**b**), pro-angiogenic monocyte/macrophage markers (*Tek/Tie2*, *Mrc1*, and *Mrc2*) (**c**), and pro-inflammatory cytokine (*Il6*) (**d**) in the cornea. The mRNA levels are presented as fold changes relative to the levels in control eyes which had not received injury or treatment. Mean values ± SD are shown from two independent experiments. Each circle depicts the data from an individual mouse. *p < 0.05, **p < 0.01, ***p < 0.001, ****p < 0.0001, as analyzed by one-way ANOVA with Tukey’s test or by Kruskal–Wallis test with Dunn’s multiple-comparisons test (*Tek* in c)

We next assessed the expression of angiogenesis-related molecules in the cornea. RT-qPCR showed that the mRNA level of *Cd31* encoding a panendothelial marker CD31 was increased in sutured corneas and significantly reduced by aflibercept (Fig. 2a). Similarly, mRNA levels of *Vegfc* and *Angpt1*, which encode major growth factors for lymphatic vessels [23], were upregulated in response to suture injury and downregulated following aflibercept treatment (Fig. 2b). In contrast, the level of *Vegfa* mRNA was furthermore increased by aflibercept (Fig. 2b).

We also evaluated the levels of *Tek/Tie2*, which encodes the angiopoietin-1 receptor, as well as *Mrc1* and *Mrc2*, which encode markers for pro-angiogenic monocytes/macrophages. We chose to evaluate these markers because, in our previous study, we discovered a strong positive correlation between the mRNA levels of *Tek/Tie2*, *Mrc1*, and *Mrc2* and the degree of corneal neovascularization [20]. The mRNA levels of *Tek/Tie2*, *Mrc1*, and *Mrc2* were all elevated in the cornea by sutures and significantly repressed by aflibercept (Fig. 2c). Similar results were obtained with the mRNA levels of the pro-inflammatory cytokine, *Il6* (Fig. 2d).

Taken together, the results suggested that subconjunctival aflibercept injection inhibited the development of corneal neovascularization by downregulating the production of angiogenic and inflammatory factors and suppressing pro-angiogenic monocytes/macrophages, in addition to directly blocking VEGF-A signaling.

### Subconjunctival aflibercept reduces VEGFR-3^*+*^ CD11b ^*+*^ cells in the cornea, blood, and DLNs

**Fig. 3:**
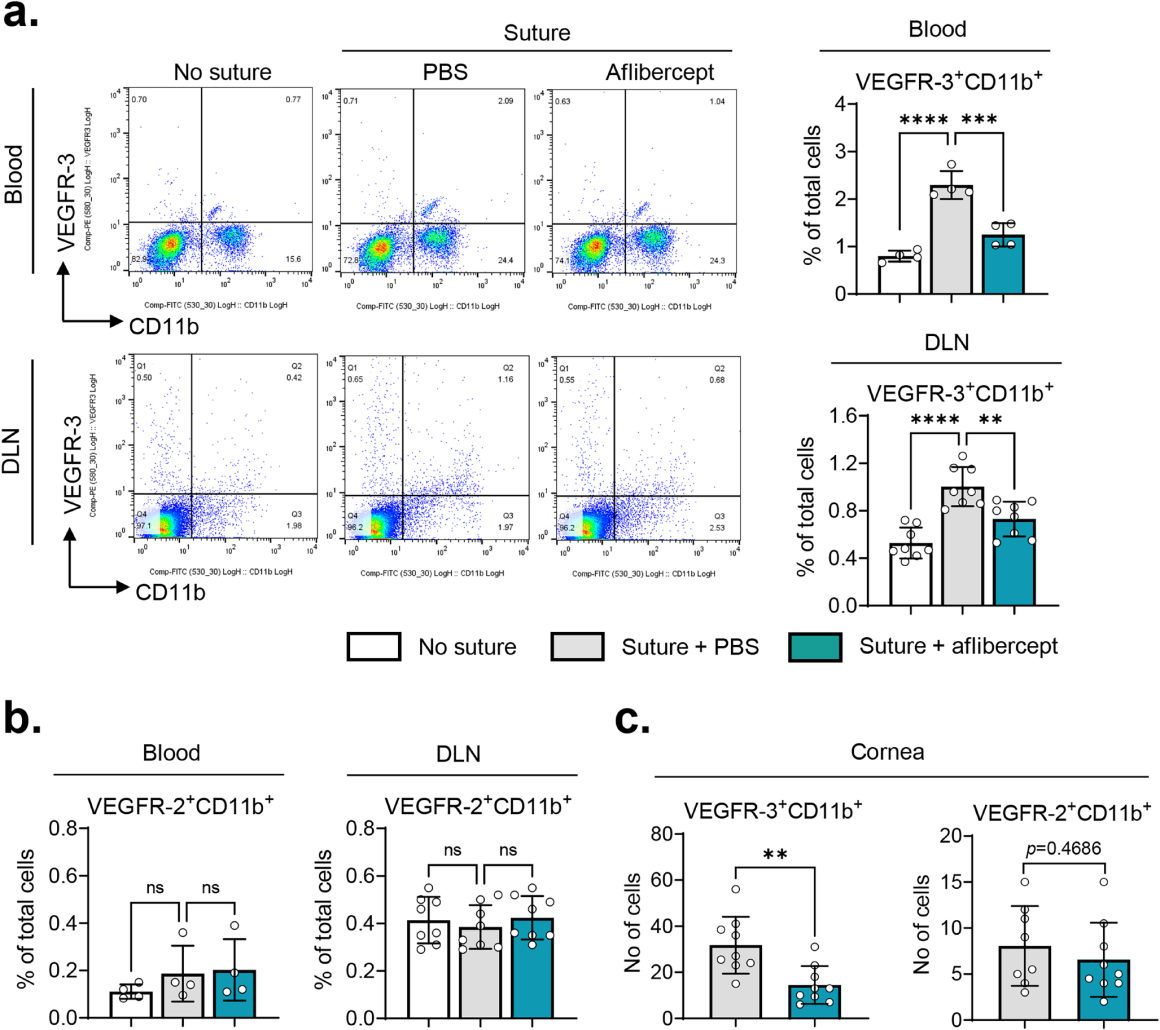
Subconjunctival aflibercept reduces the percentage of circulating and cornea-infiltrating VEGFR-3^+^CD11b^+^myeloid cells. **a, b** Representative and quantitative flow cytometry results for VEGFR-3^+^CD11b^+^ cells (**a**) or VEGFR-2^+^CD11b^+^ cells (**b**) in the blood and DLN. (**c**) Enumeration of VEGFR-3^+^CD11b^+^ cells or VEGFR-2^+^CD11b^+^ cells in the corneal flat mounts immunostained with CD11b, VEGFR-2, or VEGFR-3. Mean values ± SD are shown from two independent experiments. Each circle depicts the data from an individual mouse. **p < 0.01, ***p < 0.001, ****p < 0.0001, ns: not significant, as analyzed by one-way ANOVA with Tukey’s test (**a, b**) or by unpaired *t*-test (**c**)

Having observed the downregulation of pro-angiogenic monocyte/macrophage markers in the cornea following aflibercept treatment, we proceeded to examine the population of VEGFR-expressing myeloid cell, which are known to be highly angiogenic [24, 25]. Flow cytometric analysis demonstrated that the percentage of VEGFR-3^+^CD11b^+^ cells was increased in the blood and DLNs seven days after the placement of corneal sutures (Fig. 3a). Notably, subconjunctival injection of aflibercept significantly reduced the percentage of VEGFR-3^+^CD11b^+^ cells in both blood and DLNs (Fig. 3a). However, neither corneal sutures nor subconjunctival aflibercept injection had an impact on the percentage of VEGFR-2^+^CD11b^+^ cells in the blood or DLNs (Fig. 3b). Similar findings were observed in the cornea. The number of VEGFR-3^+^CD11b^+^ cells infiltrating the cornea was significantly reduced by subconjunctival aflibercept treatment, while the number of VEGFR-2^+^CD11b^+^ cells remained unaffected (Fig. 3c. Supplementary Fig. 1).

Therefore, the results demonstrated that subconjunctival aflibercept administration led to decreases in both cornea-infiltrating and circulating VEGFR-3^+^CD11b^+^ cells.

## Discussion

Our study yielded two key observations. First, the treatment with subconjunctival aflibercept injection was effective in inhibiting corneal angiogenesis. Second, subconjunctival aflibercept downregulated the VEGF-C signaling and reduced both circulating and cornea-infiltrating VEGFR-3^+^CD11b^+^ myeloid cells.

Aflibercept is a fusion protein consisting of an IgG backbone fused to extracellular VEGFR sequences of VEGFR-1 and VEGFR-2. As a decoy receptor, aflibercept binds to VEGF-A, VEGF-B, and PIGF (placental growth factor) with higher affinity than their native receptors, VEGFR1 and VEGFR2, effectively blocking the activity of VEGF-A, VEGF-B, and PIGF [1, 2]. Especially, the binding and activation of VEGF-A and VEGFR-2 play key roles in hemangiogenesis. Thus, the inhibitory activity of aflibercept on VEGF-A/VEGFR-2 signaling is the mechanism of action of aflibercept in treating pathologic neovascularization. In the context of corneal neovascularization, recent studies, along with our study, have demonstrated that subconjunctival, topical, or intracorneal administration of aflibercept effectively inhibits corneal new vessels in animal models of high-risk corneal transplantation or suture-induced corneal neovascularization [18, 26, 27].

One interesting finding of our study is that the mRNA level of *Vegfa* in the cornea was significantly upregulated, while mRNA levels of *Vegfc* and *Angpt1* were suppressed, after subconjunctival aflibercept treatment. A similar phenomenon was observed in a study by Puddu et al. [28], where they treated retinal pigment epithelial cells with aflibercept or ranibizumab for 24 h. They found that both aflibercept or ranibizumab increased VEGF-A and PlGF expression, while decreasing the expression and secretion of VEGF-C. The increased VEGF-A expression in the cornea or retinal pigment epithelial cells treated with VEGF-A inhibitors, such as aflibercept or ranibizumab, might be a result of compensatory responses of the cells to counter the lack of VEGF-A. Furthermore, the downregulation of VEGF-C by aflibercept, a VEGF-A inhibitor, suggests the complex regulatory mechanisms involving the VEGF family members in response to anti-VEGF treatments. Further research is needed to fully understand these intricate interactions.

Another notable observation from our study is that subconjunctival aflibercept treatment reduced both cornea-infiltrating and circulating VEGFR-3^+^CD11b^+^ cells after suturing injury, whereas VEGFR-2^+^CD11b^+^ cells were not affected by either suturing or aflibercept. VEGFR-expressing CD11b^+^ monocytes, also termed myeloid angiogenic cells, have been shown to be highly angiogenic in previous studies [24, 25]. Indeed, our group previously showed that many cornea-infiltrating CD11b^+^ cells in sutured corneas were monocytes/macrophages expressing VEGFR-3 [20]. Moreover, a study by Chung et al. demonstrated that VEGFR-3-specific signaling contributed to corneal angiogenesis in which macrophages played a major role [29]. Therefore, the suppression of VEGFR-3^+^CD11b^+^ monocytes by a VEGF-A inhibitor aflibercept, as well as *Vegfc* downregulation, might serve as one of the mechanisms underlying aflibercept’s inhibition of corneal neovascularization in our model. In line with our findings, an elegant study by Cursiefen et al. demonstrated that intraperitoneal administration of VEGF Trap, which neutralizes VEGF-A but not VEGF-C or D, completely inhibited both hemangiogenesis and lymphangiogenesis by suppressing VEGF-A-mediated recruitment of pro-angiogenic monocytes/macrophages expressing VEGF-C or D [30]. Additionally, in our study, local inhibition of VEGF-A through subconjunctival aflibercept effectively reduced the levels of systemic circulating VEGFR-3^+^CD11b^+^ cells in DLNs and blood, resulting in decreased corneal infiltration of VEGFR-3^+^CD11b^+^ cells. These findings indicate that signals released locally in the injured cornea act as chemoattractants for myeloid cells in the systemic circulation, and that the systemic mobilization of myeloid cells can be regulated by modifying the local injury signals.

In conclusion, we demonstrated that subconjunctival administration of aflibercept suppressed the expression of pro-angiogenic and inflammatory growth factors and cytokines in the cornea and reduced the number of pro-angiogenic VGFR-3^+^CD11b^+^ monocytes in the circulation and the cornea. Our results suggest that aflibercept holds promise as an effective therapeutic option for patients with corneal neovascularization. Further investigations are needed to optimize treatment regimens and conduct clinical trials in human patients.

## Electronic supplementary material

Below is the link to the electronic supplementary material.

Supplementary Material 1
