# Supplementary material for: Subconjunctival aflibercept inhibits corneal angiogenesis and VEGFR-3+CD11b+ cells
Source: Graefes Arch Clin Exp Ophthalmol. 2024 Jul 9;262(12):3881–8. doi: 10.1007/s00417-024-06560-4 (PMC11608285; doi:10.1007/s00417-024-06560-4)
Supplement: Supplementary file 1 — Supplementary Material 1 [file 417_2024_6560_MOESM1_ESM.pdf]

Article Title: Subconjunctival aflibercept inhibits corneal angiogenesis and VEGFR-3$^+$CD11b$^+$ cells

Journal name: Graefe's Archive for Clinical and Experimental Ophthalmology

Author names: Chang Ho Yoon,[1, 2] Jung Hwa Ko,[1] Hyun Ju Lee,[1] Hyun Beom Song[3, 4] and Joo Youn Oh, MD, PhD[1, 2]*

Affiliations:

[1] Laboratory of Ocular Regenerative Medicine and Immunology, Biomedical Research Institute, Seoul National University Hospital, 101 Daehak-ro, Jongno-gu, Seoul 03080, Korea

[2] Department of Ophthalmology, Seoul National University College of Medicine, 103 Daehak-ro, Jongno-gu, Seoul 03080, Korea

[3] Department of Tropical Medicine and Parasitology and Institute of Endemic Diseases, Seoul National University College of Medicine, 103 Daehak-ro, Jongno-gu, Seoul 03080, Korea

[4] Department of Biomedical Sciences, Seoul National University College of Medicine, 103 Daehak-ro, Jongno-gu, Seoul 03080, Korea

***Correspondence to:** Joo Youn Oh, MD, PhD. (jooyounoh77@gmail.com or bonzoo1@snu.ac.kr)

Supplementary Figure 1. Representative microphotographs of whole-corneal flat mounts with CD11b and VEGFR-3 immunostaining.

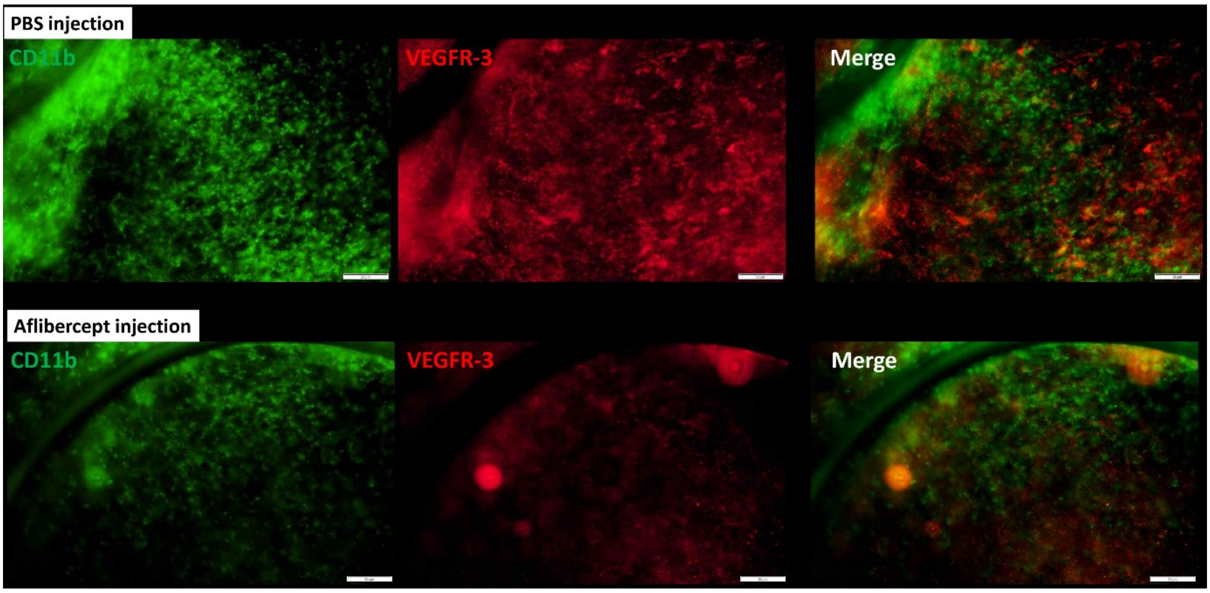

Immediately after corneal suturing injury, BALB/c mice were treated with subconjunctival injection of either PBS (5 μL) (Upper row) or aflibercept (200 μg in 5 μL; Eylea®, Regeneron Pharmaceuticals, Inc., Tarrytown, NY) (Lower row). Seven days later, the corneas were extracted and subjected to CD11b and VEGFR-3 immunostaining. Scale bar: 50 μm
